# Snail Family Members Unequally Trigger EMT and Thereby Differ in Their Ability to Promote the Neoplastic Transformation of Mammary Epithelial Cells

**DOI:** 10.1371/journal.pone.0092254

**Published:** 2014-03-17

**Authors:** Baptiste Gras, Laurent Jacqueroud, Anne Wierinckx, Christelle Lamblot, Frédérique Fauvet, Joël Lachuer, Alain Puisieux, Stéphane Ansieau

**Affiliations:** 1 Inserm UMR-S1052, Centre de Recherche en Cancérologie de Lyon, Lyon, France; 2 CNRS UMR5286, Centre de Recherche en Cancérologie de Lyon, Lyon, France; 3 LabEx DEVweCAN, Lyon, France; 4 UNIV UMR1052, Lyon, France; 5 Université de Lyon, Lyon, France; 6 Centre Léon Bérard, Lyon, France; 7 ProfileXpert, Bron, France; 8 Institut Universitaire de France, Paris, France; Northwestern University, United States of America

## Abstract

By fostering cell commitment to the epithelial-to-mesenchymal transition (EMT), SNAIL proteins endow cells with motility, thereby favoring the metastatic spread of tumor cells. Whether the phenotypic change additionally facilitates tumor initiation has never been addressed. Here we demonstrate that when a SNAIL protein is ectopically produced in non-transformed mammary epithelial cells, the cells are protected from anoikis and proliferate under low-adherence conditions: a hallmark of cancer cells. The three SNAIL proteins show unequal oncogenic potential, strictly correlating with their ability to promote EMT. SNAIL3 especially behaves as a poor EMT-inducer comforting the concept that the transcription factor functionally diverges from its two related proteins.

## Introduction

The epithelial-to-mesenchymal transition (EMT) is a latent embryonic process endowing cells with a transient migratory potential. This phenotypic switch is essential to the establishment and evolution of epithelial structures during both morphogenesis and organogenesis. Hijacking of this cell conversion mechanism has been identified as a driving force of cancer cell dissemination [1]. Since this seminal observation, a wide number of studies has confirmed that aberrant induction of master regulators of EMT, including mainly the members of the SNAIL, TWIST, and ZEB transcription-factor families, afford cancer cells motility and invasive properties [2]. While the link between EMT and metastasis is commonly accepted, a role for EMT in promoting tumor initiation has recently emerged. We and others have demonstrated that the TWIST and ZEB proteins override oncogene-induced senescence and apoptosis and cooperate with oncoproteins such as RAS and MYC to foster murine cell transformation both *in vitro* and *in vivo* [3]–[6]. Furthermore, we have demonstrated that combining a TWIST or ZEB protein with a single mitogenic oncoprotein is sufficient to promote immortalized human mammary epithelial cell (HMEC-hTERT) dedifferentiation and transformation [7].

The SNAIL transcription factors are generally considered as the gold standard EMT-inducers. In this regard, they play a central role in morphogenesis and were found as essential for mesoderm and neural crest delamination in several organisms from flies to mammals [8]–[16]. SNAIL1 (encoded by the *SNAI1* gene and previously called SNAIL) and SNAIL2 (encoded by the *SNAI2* gene and previously called SLUG) were shown a decade ago to turn down *CDH1* transcription, leading to a loss of the epithelium gatekeeper E-cadherin, and thereby to promote EMT [17]–[19]. In support of this observation, reactivation of *SNAI1* or *SNAI2* has been associated with a high risk of metastasis and a poor prognosis in different tumor progression models [20]–[22], although an inverse correlation with E-cadherin expression is not always observed [23], [24]. While SNAIL proteins are seen as potent EMT inducers associated with cancer cell dissemination, their role in tumor initiation has never been addressed. Yet detection of SNAIL1 in *in situ* ductal carcinoma, at a stage preceding cancer cell dissemination, suggests that SNAIL proteins, like the TWIST and ZEB proteins, have additional oncogenic properties [5], [6]. In support of this view, moderate upregulation of *Snai1* or *Snai2*, as induced in CombitTA-*Snai1* and CombitTA-*Snai2* transgenic mice, is associated with spontaneous development of epithelial and/or mesenchymal tumors.

The SNAIL3 (encoded by the *SNAI3* gene and previously called SMUC) transcription factor is the last member of the family to be acknowledged, originally cloned by PCR from adult skeletal muscle with degenerative primers, before being identified through *in silico* analyses [25], [26]. The protein shares with both SNAIL1 and SNAIL2 proteins a similar structural organization encompassing an N-terminal SNAG transrepression domain and a C-terminal DNA binding domain encompassing 4 to 5 zinc-fingers [26]. Expression analysis of *Snai3* expression by *in situ* hybridization during mouse embryonic development demonstrated that *Snail3* transcripts are specifically detected in skeletal muscle and thymus at a relatively late stage of mouse development [27] suggesting specific and EMT-unrelated functions of SNAIL3. In support of this conclusion, using a *Snai3*-EYFP transgenic mouse model, *Snai3* expression was confirmed to be constrained to skeletal muscle and thymus and not to EMT sites [28]. Furthermore, *Snai3* null mice do not exhibit any obvious phenotype including no evident defect in T lymphocyte development [28], while *SNAI3* transduction in hematopoietic stem cells was previously shown to favor their commitment into the myeloid lineage at the expense of the lymphoid lineage [29]. Lack of phenotype has recently been explained by demonstrating that SNAIL2 and SNAIL3 display redundant functions in regards to B and T cell differentiation. This functional redundancy is likely not restricted to lymphomagenesis, as *Snai2^−/−^ Snai3^−/−^* double knockout mice elicit a more severe phenotype than single *Snai2^−/−^* knockout, namely a stunted growth phenotype, a paucity of offspring, in addition to the previously discussed inhibition of B and T cell development [30]. Collectively, this information suggests that the three SNAIL proteins are not functionally equivalent but rather behaves as overlapping modules. SNAIL1 and SNAIL2 proteins share similar EMT-promoting functions with a different predominance in mammals and birds [12], [16], [31] and SNAIL2 and SNAIL3 are both implicated in hematopoietic stem cell fate. In line with their expression profile during embryonic development, we herein demonstrate that, SNAIL3, unlike SNAIL1 and SNAIL2, actually behaves as an inefficient EMT-inducer in immortalized but non-transformed mammary epithelial cells. We next exploit this differential efficiency to further explore the link between the cell commitment into EMT and the acquisition of neoplastic transformation-associated properties.

## Results

### *SNAI3* is aberrantly reactivated in breast cancers

**Figure 1:**
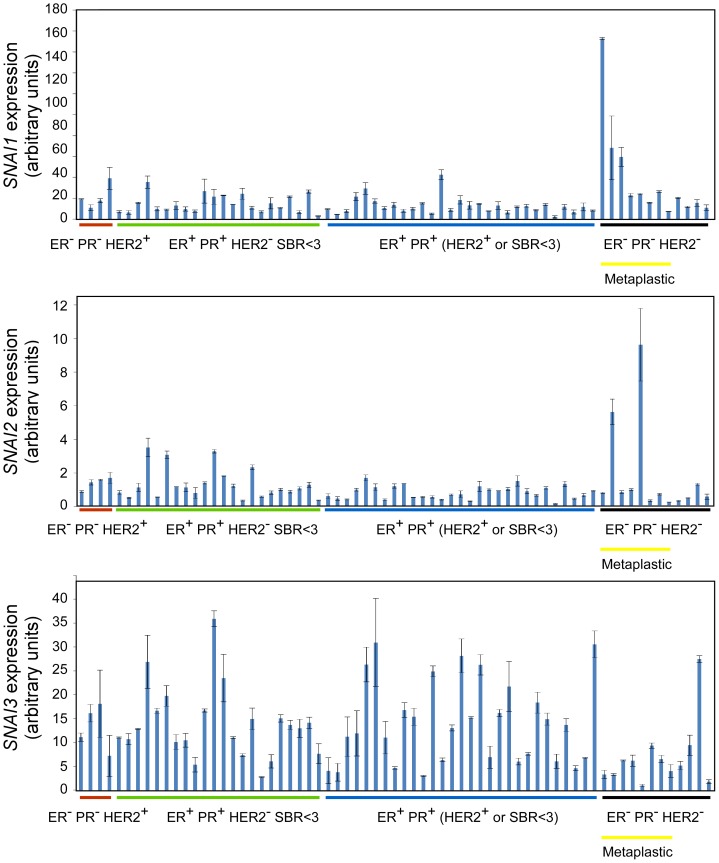
Determination by RT-qPCR of *SNAI1, SNAI2 and SNAI3* transcript levels in human primary mammary tumors. Levels expressed relatively to housekeeping gene transcripts were normalized with respect to HMEC-hTERT cells. ER/PR: expression analysis of the estrogen and progesterone receptor, + means >10% expressing cells. HER2^+/−^: amplification status of the *ERBB2* gene. SBR: Scarff-Bloom-Richardson grade. Metaplastic tumors include malpighian and sarcomatoïd carcinomas.

**Figure 2:**
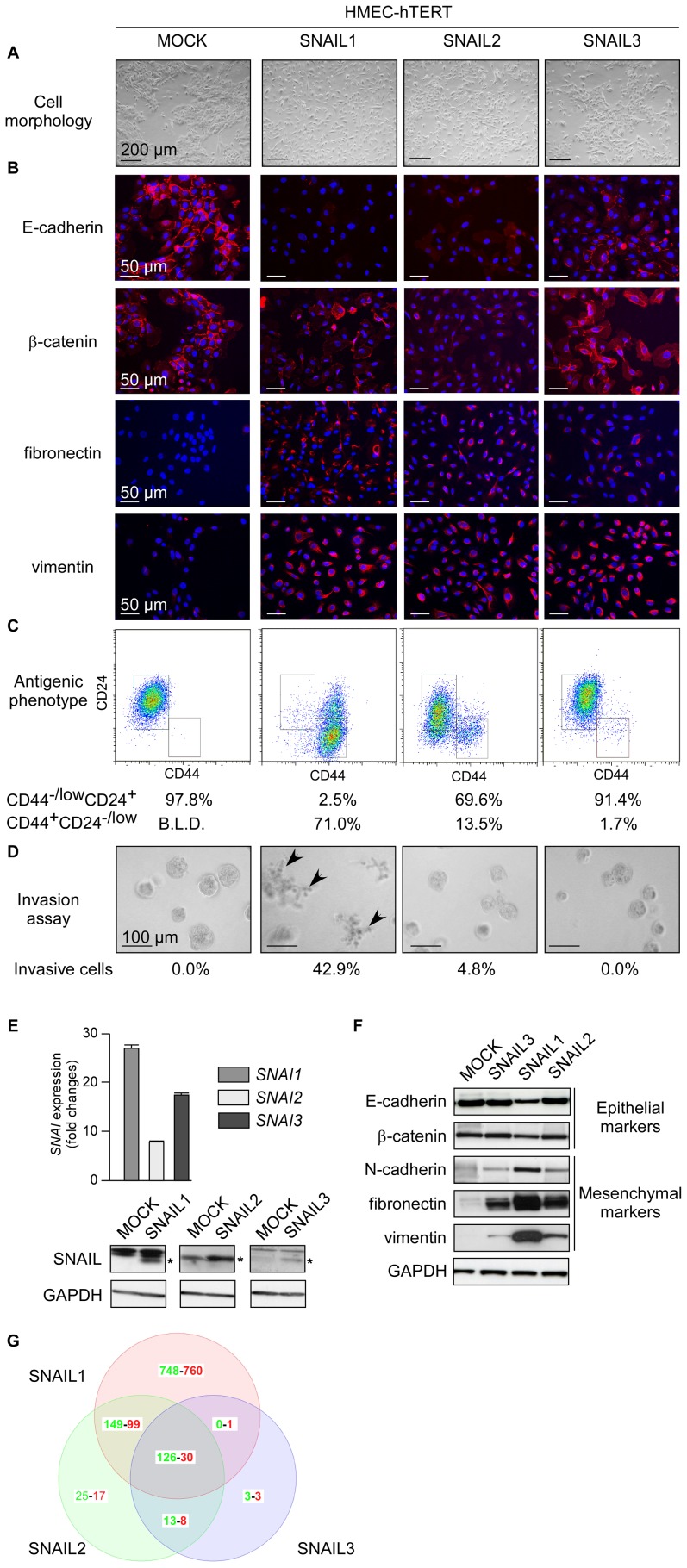
SNAIL proteins act with different potency to promote human mammary epithelial cell commitment into EMT. HMECs were infected with the *SNAI* retroviral constructs as indicated at the top. (A) Representative photomicrographs of cells obtained by phase contrast microscopy. (B) Analysis of epithelial markers (E-cadherin, β-catenin) and mesenchymal markers (fibronectin, vimentin) by immunofluorescence. (C) Analysis by flow cytometry of CD44 and CD24. Percentages of CD44^−/low^ CD24^+^ and CD44^+^CD24^−/low^ cells are indicated. B.L.D.: below the limit of detection. (D) Invasion assay. Percentages of invasive cells are indicated. Invasive cells are arrowed. (E) Upper panel: Analysis of *SNAI-*transgene expression by qRT-PCR in the corresponding transfected cell lines. Levels are expressed relatively to the housekeeping gene *HPRT1*. Lower panels: western blot analysis of SNAIL proteins. Proteins of interest are indicated by stars. (F) Analysis of epithelial and mesenchymal markers by western blotting. (G) Venn diagram showing the overlap of genes upregulated (in red) or down-regulated (in green) in SNAIL-HMEC derivatives as compared to the parental HMEC-hTERT cell line and as determined with a 1.5-fold cut-off and a p value <0.1.

While the *SNAI1* and *SNAI2* genes are reported to be frequently reactivated in numerous carcinomas (breast, esophageal, colon, kidney) [32]–[35], the status of the related *SNAI3* gene has remained unclear. To address this question, *SNAI3* expression was assessed by qRT-PCR in a cohort of primary human tumors (n = 44) encompassing four different carcinoma types (colon, lung, ESCC, kidney), as compared to healthy tissues or normal cell counterparts. *SNAI3* transcription was barely detectable in colon and lung cancers and weakly induced in ESCC and kidney cancers (data not shown), but significantly induced in primary non-metastatic breast tumors from untreated patients, as compared to immortalized mammary epithelial cells (HMEC-hTERT) (FC>10 in 58.2%, n = 67) (Figure 1). In these primary breast tumors, *SNAI1* transcription was also frequently induced (FC>10 in 68.6%, n = 67) in line with previous reports [36]–[38]. We confirmed that the *SNAI2* gene is transcriptionally active in HMEC-hTERT cells (Figure 2E) [39], an expression that remained largely unaffected in primary tumors. Consistently with this observation, *SNAI1* is commonly induced in human breast carcinoma cell lines (FC>10, 63.6%, n = 11) as compared to HMEC-hTERT cells. The *SNAI3* gene also appeared to be expressed in several human breast cancer cell lines, although the induction level was much lower (FC>5 in 36.3%, n = 11), while *SNAI2* expression remained unchanged (Figure S1). *SNAI1* and *SNAI3* induction did not associate with a specific tumor subtype and were not correlated one to each other.

In conclusion, both *SNAI1* and *SNAI3* gene expressions are induced in primary breast tumors compared to normal mammary epithelial cells, suggesting that likewise SNAIL1, SNAIL3 may also contribute to breast tumorigenesis.

### SNAIL proteins promote EMT with unequal efficiency

In the light of its different expression profile during development [27], we reasoned that SNAIL3 might differ from SNAIL1 and SNAIL2 as regards EMT induction, a difference that might be exploited to evaluate the contribution of EMT to cell transformation. To explore this possibility, immortalized mammary epithelial cells (HMEC-hTERT) were infected with SNAIL-encoding retroviral constructs (the resulting cells are hereafter referred to as SNAIL-HMECs). N-terminal tagged versions of the proteins were firstly used. Nonetheless, the few additional residues were found to annihilate SNAIL protein function likely by interfering with the N-terminal SNAG transrepression domain (data not shown). We thus sought to compare the activities of untagged versions of the SNAIL proteins, despite the difficulty to confirm equal protein expression. Examination of cell morphology and assessment of epithelial markers (E-cadherin, β-catenin) and mesenchymal markers (N-cadherin, fibronectin, and vimentin) by immunofluorescence and western blotting (Figure 2A, B, F) demonstrated that all three proteins promoted cell commitment to EMT, but with varying efficiency. SNAIL1-HMECs underwent almost complete EMT. Although expressing a residual amount of E-cadherin, they displayed a fibroblastic morphology and invasive properties. By comparison, SNAIL2- and SNAIL3-HMECs were found to be only partially committed to the transdifferentiation program, as demonstrated by weakened cell-cell contacts and co-expression of epithelial and mesenchymal markers (Figure 2). Strikingly, SNAIL2- and SNAIL3-HMECs lacked invasive properties (Figure 2D). In line with the role of EMT in cell dedifferentiation [40], [41], 71.0% of the SNAIL1-, 13.5% of the SNAIL2-, and 1.7% of the SNAIL3-HMECs displayed a stem-cell-like CD44^+^CD24^−/low^ antigenic phenotype. To further strengthen our conclusions, gene expression profiles of the HMEC-derived cell lines were established. Comparison of these gene expression profiles with the recently established EMT-associated signature [42] confirmed the gradient of efficiency of the SNAIL proteins in promoting EMT (Table S1). Interestingly, while the expression of no less than 404 genes was found to respond - in similar fashion - to both SNAIL1 and SNAIL2, only 156 of them appeared to be modulated by SNAIL3 (Figure 2G). In keeping with a previous study [43], SNAIL1/2 production appeared associated with decreased expression of luminal differentiation markers and with activation of the TGFβ pathway. To rule out the possibility that the inefficiency of SNAIL3 to promote EMT results of protein instability, C-terminal tagged versions of SNAIL1 and SNAIL3 were generated. Despite a higher level of expression and a correct nuclear localization (Figure S2B–D), SNAIL3 still remained inefficient in promoting EMT in HMEC-hTERT cells, as judged by the cell morphology (Figure S2A) and the expression of epithelial and mesenchymal markers (Figure S2C, D). As EMT induction by SNAIL1 is mediated by the direct transcriptional repression of *CDH1* (the E-cadherin encoding gene), we next compared the efficiency of SNAIL1 and SNAIL3 to repress the transcriptional activity of the *CDH1* promoting sequences, as assessed in a reporter assay [19]. While SNAIL1 as expected successfully annihilated reporter expression in an E-box integrity dependent manner, SNAIL3 failed to do so (Figure S2E). Collectively, these experiments demonstrated that EMT-induction is not the primary function of SNAIL3, as expected from its embryonic expression pattern [27].

**Figure 3:**
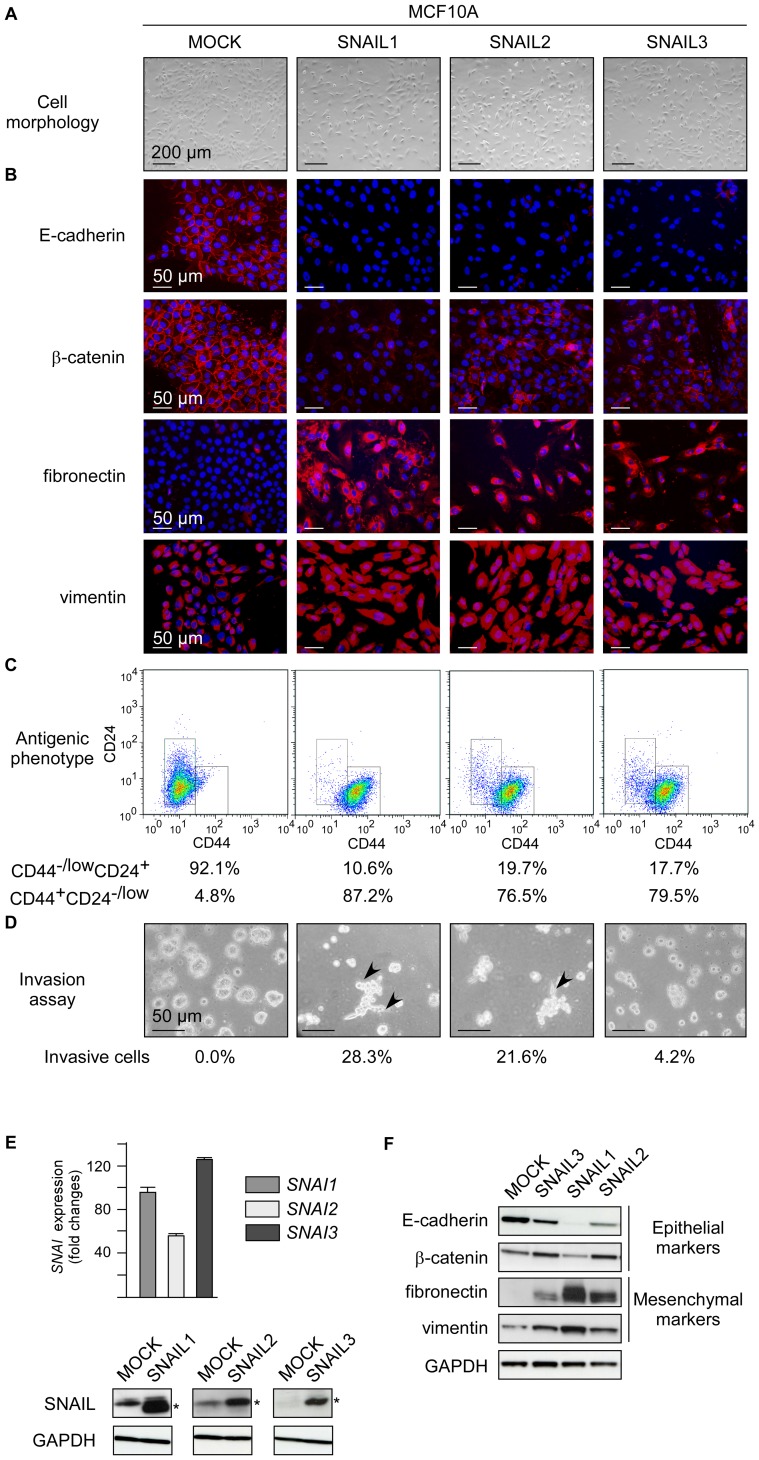
SNAIL proteins promote MCF10A commitment to EMT. MCF10A cells were infected with constructs encoding SNAIL proteins as indicated at the top and characterized. (A) Representative photomicrographs of cells obtained by phase contrast microscopy. (B) Analysis of epithelial (E-caherin, β-catenin) and mesenchymal (fibronectin, vimentin) markers by immunofluorescence. (C) Analysis by flow cytometry of CD44 and CD24. (D) Invasion assay. Percentages of invasive cells are indicated. (E) Upper panels: analysis of ectopic *SNAI* expression by qRT-PCR. Transcript levels are expressed with respect to transcripts of the *HPRT1* housekeeping gene. Lower panels: western blot analysis of SNAIL proteins. Proteins of interest are indicated with stars. (F) Analysis of epithelial and mesenchymal markers by western blotting.

The immortalized MCF10A breast cell line is more responsive to EMT-promoting cytokines than HMEC-hTERT cells, suggesting that active intracellular pathways/factors make them prone to commit into this transdifferentiation program (data not shown). We thus evaluated whether SNAIL3 remained inefficient in inducing EMT in such a favorable cellular context or may cooperate with such pathways/factors to trigger EMT. The phenotypic switch was actually detectable with all three SNAIL proteins (Figure 3A, B, E). All MCF10A derivatives predominantly displayed a stem-cell-like antigenic phenotype (Figure 3C). Nonetheless, a gradient of EMT-promoting activity remained detectable (SNAIL1>SNAIL2>SNAIL3), as demonstrated by assessment of epithelial and mesenchymal markers (Figure 3F), gene expression profiles (Table S1), and cell invasive properties (Figure 3D). Despite some inefficacy in triggering EMT on its own, SNAIL3 may thus take part in EMT-promoting interactomes [42], [44] and thereby to some extent contribute to the cell reprogramming.

Ectopic expression of *SNAI* mRNAs in HMEC-hTERT and MCF10A cells provided syngenic cell lines that only differ by their commitment rate in EMT. We thus sought to next take profit of these cellular models to explore the contribution of EMT in the acquisition of some malignant properties.

### SNAIL proteins can endow immortalized mammary epithelial cells with malignant properties

**Figure 4:**
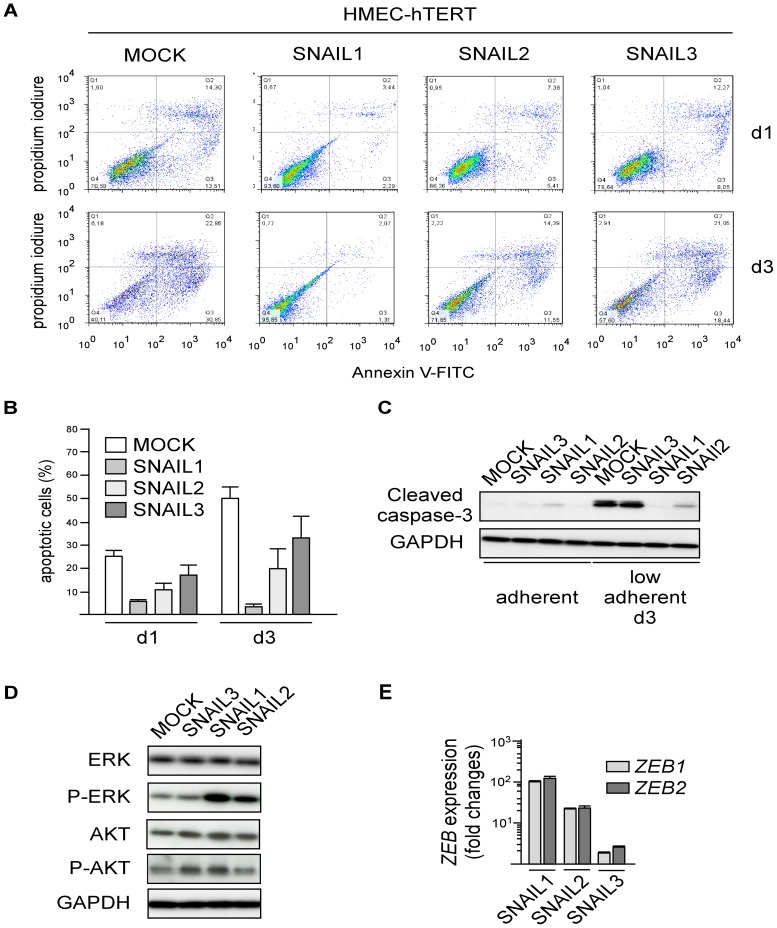
SNAIL proteins confer a survival advantage to HMEC-hTERT cells under low-adherence conditions. (A) Upper panels: HMEC-derived cell lines were cultured in ultra-low attachment dishes for different periods of time as indicated on the right. The cells were then stained with annexin V-FITC and propidium iodide and analyzed by flow cytometry. (B) Percentages of apoptotic cells (including Annexin V^+^/PI^−^ and Annexin V^+^/PI^+^ cells) are indicated as means ±SD of triplicate experiments. (C) Analysis of the cleaved caspase-3 fragment by western blotting. (D) Examination by western blotting of the status of the ERK and AKT pathways. P-ERK and P-AKT stand for phospho-T202, Y204 ERK1/2 and phospho-S473 AKT respectively. (E) Expression analysis of *ZEB1* and *ZEB2* in HMEC-hTERT cells ectopically expressing either *SNAI1*, *SNAI2* or *SNAI3*. Levels expressed relatively to the housekeeping *HPRT1* gene transcripts were normalized with respect to HMEC-hTERT cells ±SD of triplicates.

**Figure 5:**
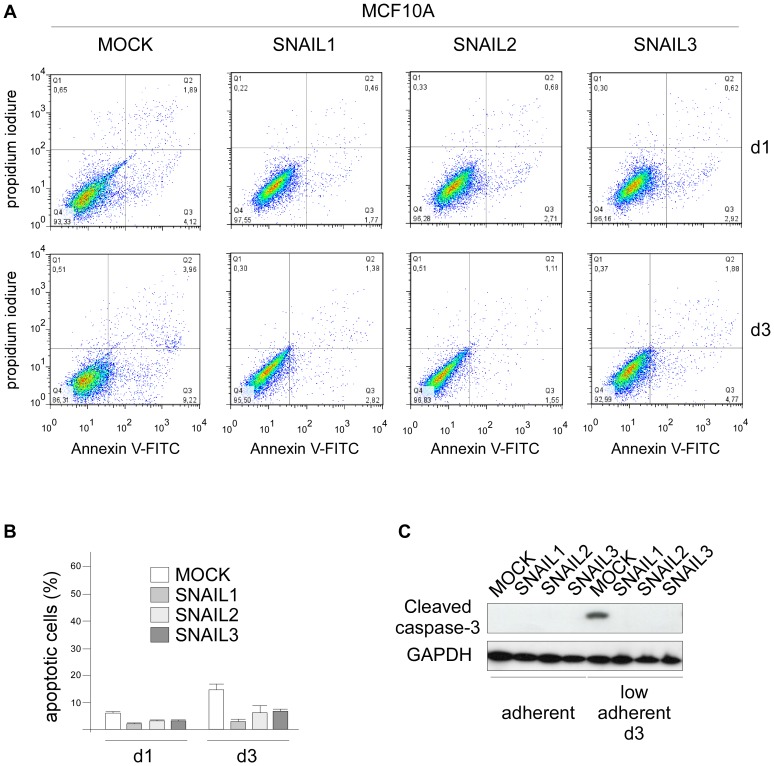
SNAIL proteins confer a survival advantage to MCF10A cells under low-adherence conditions. (A) MCF10A cells infected with SNAIL-protein-encoding constructs, as indicated at the top, were cultured in ultra-low attachment dishes for different periods of time as indicated on the right. Cells were then stained with annexin V-FITC and propidium iodide and analyzed by flow cytometry. The results shown are representative of three independent experiments. (B) Histogram showing percentages of apoptotic cells (including annexin V^+^/PI^−^ and annexin V^+^/PI^+^ cells) means with SD of triplicate experiments. (**C**) Analysis of cleaved caspase-3 fragment by western blotting.

**Figure 6:**
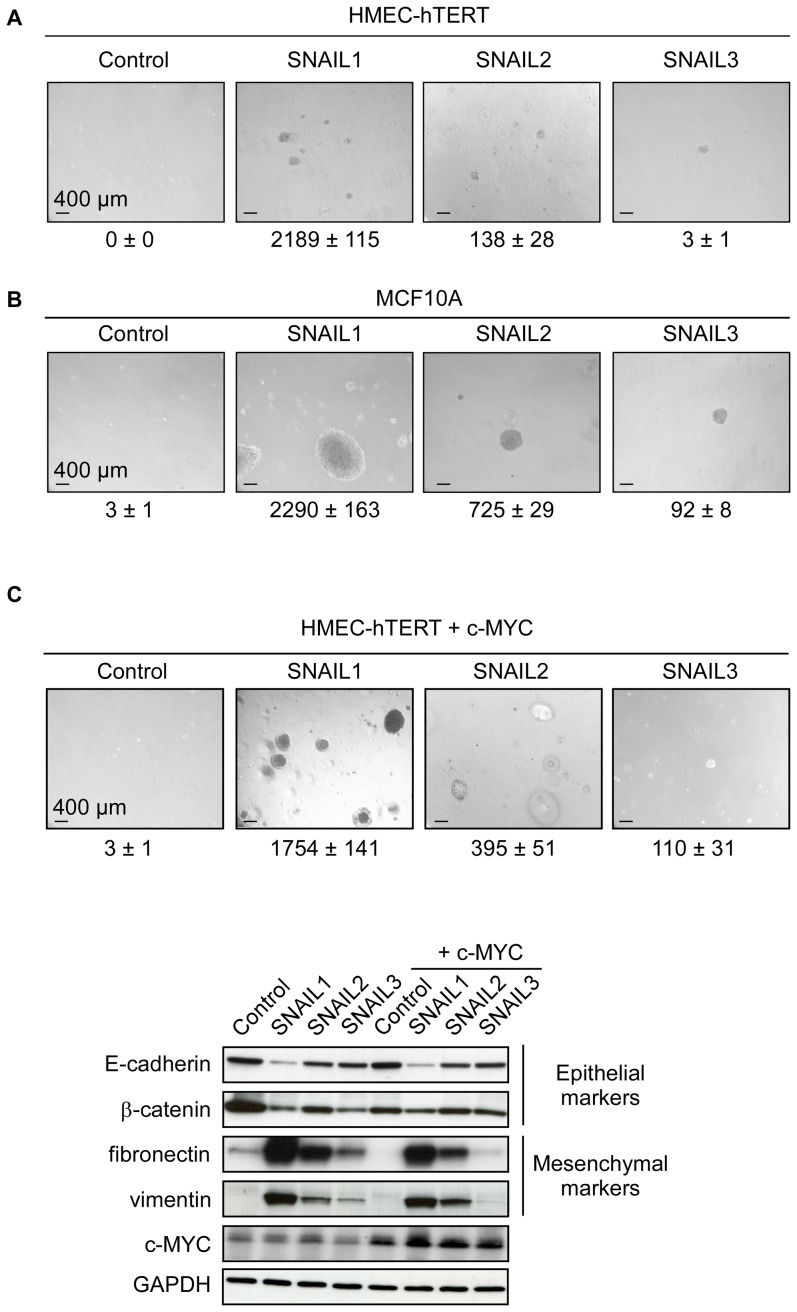
SNAIL proteins are sufficient to promote mammary epithelial cell transformation. (A) HMECs were transfected with the SNAIL-protein-encoding construct as indicated at the top and their transformation potential was assessed in a soft-agar colony assay. Numbers of colonies are means of triplicate counts. (B) MCF10A cells were similarly transfected with SNAIL-encoding constructs and their transformation potential assessed in a soft-agar colony assay. Numbers of colonies are means of triplicate counts. (C) HMECs were sequentially transfected with the SNAIL and c-MYC protein-encoding construct as indicated at the top and their transformation potential was assessed in a soft-agar colony assay (upper panels). Numbers of colonies are means of triplicate counts. The analysis of epithelial and mesenchymal markers by western-blotting demonstrated that the exacerbated growth of colonies did not correlate with a further commitment into EMT (lower panels).

To invade the lumen ducts or colonize secondary sites, cancer cells have to survive detachment from the extracellular matrix, an event committing normal cells to a cell-death program known as anoikis. EMT, by down-regulating E-cadherin expression, is known to render cells resistant to anoikis [45], [46]. We thus examined whether the extent to which a SNAIL protein promotes EMT might affect its ability to protect cells under stress. To this end, we cultured SNAIL-HMEC and SNAIL-MCF10A cells in ultra-low-attachment culture dishes for different periods of time and monitored cell death either by FITC-Annexin V/PI labeling or by measuring the level of activated caspase-3. As suspected, the three SNAIL proteins were indeed found to protect HMEC-hTERT cells from anoikis with different efficiency, and the degree of protection observed (SNAIL1>SNAIL2>SNAIL3, Figure 4A–C) paralleled their ability to promote EMT. It also paralleled their ability to activate pathways (AKT and MAPK) (Figure 4D) and downstream genes (*ZEB1/2*) (Figure 4E) reported to determine the capacity to survive detachment from the extracellular matrix [47], [48]. In MCF10A cells, where all three SNAIL proteins successfully trigger EMT (Figure 3), they were found to protect the cells similarly from anoikis (Figure 5). As we previously demonstrated that EMT facilitates human mammary epithelial cell transformation [7], we next assess the SNAIL oncogenic potential by performing soft agar colony assays. SNAIL1 was found to stimulate potently the transformation of HMEC-hTERT cells. While SNAIL2 caused the appearance of a few colonies on agar, SNAIL3 showed practically no transforming potential (Figure 6A). Forced production of SNAIL proteins in MCF10A likewise triggered cell transformation with a gradient of efficiency mirroring the gradient of ability to trigger EMT (Figure 6B). Colonies were found to be larger when experiments were performed in MCF10A, suggesting that these cells display a proliferative advantage when cultured in such low-adherence conditions. As MCF10A display an amplification of *MYC* [49], we thus evaluated whether combining SNAIL proteins with c-MYC in HMEC-hTERT cells may similarly improve the colony growth. As shown in Figure 6C, adjoined expression of c-MYC affords cells a growth advantage demonstrating a synergistic effect of c-MYC and SNAIL proteins in promoting mammary epithelial cell transformation. Noticeably, the malignant transformation remained partial as cells remained devoid of a tumorigenic potential, when subcutaneously xenografted into *nude* mice (data not shown). The synergistic effects of c-MYC and SNAIL proteins also did not further engage cells into EMT, as demonstrated by the expression analysis of epithelial and mesenchymal markers by western blotting (Figure 6C).

## Discussion

A growing body of evidence supports the view that EMT-inducing transcription factors play a dual role during tumor progression, promoting malignant conversion on the one hand and the metastatic cascade in the other [2]. While the pro-metastatic properties of SNAIL proteins are well documented, their potential contribution to tumor initiation has never been investigated. To address this issue, we have estimated the oncogenic potential of SNAIL proteins in immortalized human mammary epithelial cells. We have included in our analysis SNAIL3, whose gene shows aberrant expression in human primary breast tumors (Figure 1). Obviously, the lower level of *SNAI3* transcript observed in breast cancer cell lines than in tumors suggests that part of the signal observed in patient samples is attributable to the stromal compartment. We have unfortunately failed to address this hypothesis, as the commercially available anti-SNAIL3 antibody is not specific enough. It is worth noting that the SNAIL1 protein has been detected in both the epithelial and stromal compartments of breast ductal carcinomas [39]. By infecting non-transformed mammary epithelial cells with *SNAI* retroviral expression constructs, we have highlighted a direct correlation between the ability of a protein to cause the cells to commit to EMT and its ability to promote cell survival and proliferation in the absence of adhesion, a feature of cancer cells [50]. SNAIL3 appears much less active than SNAIL1 and SNAIL2 in HMEC-hTERT cells (Figure 2). Reproducing experiments with C-terminal tagged versions of SNAIL1 and SNAIL3 confirmed that the differential activity of the two proteins relied on neither dissimilar protein stability nor on inappropriate subcellular localization (Figure S2). At this stage, however, we cannot exclude the possibility that essential signals for SNAIL3 activity (post-translational modifications) might be absent in HMECs. As SNAIL3-MCF10A cells did commit to transdifferentiation, albeit incompletely (Figure 3), these requested activation steps may occur in that cellular context. Alternatively and more likely, EMT induction does not constitute the primary function of SNAIL3. In support of this conclusion, SNAIL3 is undetectable at EMT sites during embryonic development [27]. Nonetheless, SNAIL3 may take part in complex interactomes generated by EMT-inducers and signaling pathways [42], [44] and thereby contributes to some extent to EMT induction, as observed in MCF10A.

EMT-induction associates with a gain of some malignant properties. In line with previous reports, commitment into the transdifferentiation program affords cells a resistance to anoikis [45]–[47]. We herein demonstrate that SNAIL proteins, strictly correlating with their ability to promote EMT, additionally afford cells a proliferation advantage in low-adherent conditions. Based on their differential efficiency in promoting the malignant transformation of MCF10A and HMEC-hTERT, we next explored a potential cooperation with c-MYC and actually found that SNAIL proteins cooperate with the mitogenic oncoprotein in promoting colony formation in a soft-agar assay. This observation provides a rationale to the recent detection of the SNAIL1 protein in *in situ* ductal carcinoma, at a stage where tumors do not spread and further strengthens our recent demonstration of a role of EMT in facilitating the malignant transformation of epithelial cells [7].

## Materials and Methods

### Expression vectors

The cDNAs encoding human wild-type SNAIL1 (GenBank NM_005985), SNAIL2 (GenBank NM_003068), and SNAIL3 (GenBank NM_178310) were generated by PCR and subcloned into the pBabe-Puro (Addgene) or pPRIP-Puro retroviral vector [51]. HA-tag was inserted in the C-terminus of SNAIL1 or SNAIL3 by PCR-mutagenesis. The c-MYC pBabe retroviral construct was generously provided by Martin Eilers (Würzburg University, Germany).

### Cell culture

Primary HMECs were provided by Lonza and immortalized by expression of the gene encoding the catalytic subunit of the telomerase hTERT. Derivatives were cultured in 1∶1 Dulbecco's Modified Eagle's Medium (DMEM)/HAMF12 Glutamax medium (Invitrogen) supplemented with 10% FBS (Invitrogen), 0.5% penicillin-streptomycin (Invitrogen), 0.5% gentamycin (Invitrogen), 10 ng/ml human epidermal growth factor (EGF) (PromoCell), 0.5 mg/ml hydrocortisone (Sigma), and 10 mg/ml insulin (Actrapid). MCF10A cells were provided by the ATCC and cultured as described in [52]. Human cancer cell lines were provided by the ATCC and cultured according to the supplier's recommendations. Anoikis-resistance assays were performed by seeding 2×10^5^ cells into 6-well Ultra-Low Adherence dishes (Corning) for 1 or 3 days. The cells were then collected by centrifugation. Aggregates were dissociated by incubating the cells for 10 min with TrypLE Express at 37°C and the cells were used for further analysis.

### Retroviral infection

Retroviral particles were generated by PEI transfection of the amphotrophic GP293 or the ecotrophic PlatE packaging cell line with the retroviral vector pBabe or pPRIP into according to the manufacturer's recommendations (Euromedex). Viral stocks were harvested two days post-transfection, filtered (pore size: 0.45 μm), diluted 1∶2 and placed in contact with cells for 10–12 h in the presence of 8 μg/ml polybrene. Sequential infections were spaced by a 48 h period of time. Selection was initiated 24 h post-infection (or post-second infection) with 0.5 μg/ml puromycin or 100 μg/ml neomycin and continued for 7 days.

### Immunoblot analysis

Cells were washed twice with a PBS- 0.5% EGTA solution and lysed in RIPA buffer (150 mM NaCl, 1% NP40, 100 mM Tris pH 7.5, 0.1% SDS, 0.5% DOC, 1 mM EGTA) supplemented with complete protease (Roche) and phosphatase inhibitors (Sigma). After clarification by centrifugation at 14 000 rpm for 20 min at 4°C, cell extracts were subjected to SDS-PAGE. Proteins were revealed with mouse monoclonal anti-E-cadherin (clone 36, Becton Dickinson), anti-β-catenin (clone 14, Becton Dickinson), anti-fibronectin (clone 10, Becton Dickinson), anti-vimentin (clone V9, Dako), anti-N-cadherin (Becton Dickinson), anti-phospho-ERK1/2 (9106S, Cell Signaling), anti-GAPDH (6C5, Biodesign), rabbit monoclonal anti-active caspase 3 (ab32042, Abcam), or rabbit polyclonal anti-SNAIL1 (ab17732, Abcam), anti-SNAIL2 (G-18/SC-10436, Tebu-bio), anti-SNAIL3 (HPA016757, Sigma), anti-ERK1/2 (#9102, Cell Signaling), anti-AKT (8272, Cell Signaling), anti-phospho-AKT (Ser473) (#4058, Cell Signaling), anti-c-MYC A14 (sc-789, Santa Cruz Biotechnology), anti-HA Y11 (Santa-Cruz) and horseradish-peroxidase-conjugated secondary antibodies (Dako). Antigen-antibody complexes were revealed with a reagent for western blotting (Santa Cruz).

### Analysis of SNAI3 expression in human samples and cancer cells

Primary tumor samples were obtained though the Biological Resource Center of the Centre Léon Bérard with the agreement of the reviewal board of the Centre Léon Bérard. Samples were used with the patient's written informed consent. The present study was approved by the reviewal board of the Centre Léon Bérard.

RNA was extracted from tumors and cell lines (Figures 1 and S1) with the RNeasy Mini Kit (Qiagen) and its quality checked with the Agilent RNA 6000 Nano kit (Agilent Technologie). cDNAs were produced from 100 ng RNA with the High Capacity RNA-to-cDNA kit (Foster City, USA). Amplification was performed on a TaqMan Low Density Array (TLDA) with the Taqman Universal PCR Master Mix (Applied Biosystem). *ACTB*, *HPRT1*, and *ARNT* were used as internal controls. List of assays ID: *SNAI1* Hs00195591_m1; *SNAI2* Hs00161904_m1, *SNAI3* Hs01018996_m1, *ACTB* Hs99999903_m1, *HPRT1* Hs01003267_m1, *ARNT* Hs00234048_m1.

### Gene expression analysis in established human cell lines

Total RNA (Figures 2, 3 and 4) was extracted with the RNeasy minikit (74106, Quiagen) according to the manufacturer's recommendations. Reverse transcription was performed from 1 μg total RNA with the Dynamo cDNA synthesis kit (F-470L, Thermo Scientific). The reverse transcription product was diluted 1∶10 and used as cDNA template for qPCR analysis. SYBR green quantitative PCR was carried out in a CFX96 Real-time PCR detection system (Biorad). PCR mixtures contained SsoAdvanced SYBR Green supermix (1725264, Biorad) and 200 nM primers. The *HPRT1* housekeeping gene was used for normalization. Real-time PCR intron-spanning primers were designed with the Primer3 software. The following combinations of primers were used: *SNAI1* 5′-GCTGCAGGACTCTAATCCAGA-3′ and 5′-ATCTCCGGAGGTGGGATG-3′, *SNAI2* 5′-TGGTTGCTTCAAGGACACAT-3′ and 5′-GTTGCAGTGAGGGCAAGAA-3′; *SNAI3* 5′-CCACAGGGTCCCCAACTAC-3′ and 5′-GAGCAGGCACCATTGATTTC-3′; *ZEB1* 5′-AACTGCTGGGAGGATGACAC-3′ and 5′-TCCTGCTTCATCTGCCTGA-3′, *ZEB2* 5′-AAGCCAGGGACAGATCAGC-3′ and 5′-GCCACACTCTGTGCATTTGA-3′, *HPRT1* 5′-TGACCTTGATTTATTTTGCATACC-3′ and 5′-CGAGCAAGACGTTCAGTCCT-3′.

### Microarray analysis

Microarray processing and data analysis were performed at the ProfileXpert core facility (Lyon, France). Gene expression profiles were analyzed with a whole human genome microarray containing 47231 probes (HumanHT-12 v4 Expression BeadChip; Illumina Inc., USA). Total RNA (500 ng) was amplified and biotin-labeled with the Illumina TotalPrep™ RNA Amplification Kit (Ambion Inc., USA). Hybridization was performed with 750 ng biotin-labeled cRNA on each BeadChip. The standard Illumina scanning protocol was used to scan the arrays with the iScan (Illumina Inc., USA). Data were normalized by quantile normalization with Genome Studio Software 2010 (Illumina Inc., USA). The complete set of raw and normalized files is available at the GEO database under accession number GSE40690. Data were analyzed with tools in Partek Genomic Suite 6.6 software (Partek Inc., St. Louis, MO). One-way ANOVA was performed to compare the different groups with controls. Gene lists were filtered with a fold-change cutoff of 1.5 and p<0.1. We used the Venn diagram to visualize relationships between the created gene lists.

### Antigenic profile analysis

Cells detached with TrypLE-Express (12605-010, Invitrogen) were counted and then incubated in blocking solution (PBS containing 0.5% BSA (Sigma)) at 4°C for 30 min. Cell distributions were determined with FITC-CD44 G44-26 (BD Pharmingen), and PE-CD24 ML5 (BD Pharmingen) monoclonal antibodies and the FACScan Calibur (Becton Dickinson) and analyzed with the FlowJo software.

### Annexin/PI analysis

Cells collected by centrifugation were counted and then incubated with annexin V-FITC in binding buffer 1X (10 mM Hepes/NaOH pH 7, 140 mM NaCl, 2.5 mM CaCl_2_) for 10 min in the dark according to the manufacturer's protocol (Abcyss). DNA staining was then performed with propidium iodide (0.6 μg/ml) for 10 min. For each condition, 10^4^ events were recorded by FACS (FACScan Calibur, Beckman Dickinson) and results were analyzed with the FlowJo software.

### Immunofluorescence

10^4^ cells were seeded onto an 8-well Lab-TekII chamber slide, fixed in 3.7% formaldehyde solution (Sigma), and permeabilized in 0.1% Triton 100X (Sigma), PBS buffer at room temperature for 10 min. The cells were then washed 3 times with PBS and incubated for 1 h with 10% horse serum in PBS blocking solution. The cells were incubated overnight at 4°C with murine monoclonal anti-E-cadherin clone 36, (Becton Dickinson), anti-β-catenin clone 14 (Becton Dickinson), anti-fibronectin clone 10 (Becton Dickinson), anti-vimentin clone V9 (Dako), or with a polyclonal rabbit anti-HA Y11 (Santa-Cruz) primary antibody, washed in PBS, and then incubated for 1 h at room temperature with a goat anti-mouse Alexa Fluor 533 secondary antibody (A21422, Invitrogen) or with a mouse anti-rabbit Alexa Fluor 555 secondary antidody (A21428, Invitrogen). After extensive washes in PBS, the nuclei were stained with 5 mg/ml Hoechst for 10 min and mounted with Fluoromount-G (SouthernBiotech). All matched samples were photographed with an immunofluorescence microscope (Leica) and identical exposure times.

### Invasion assay

Invasion assay consisted in culturing 5×10^3^ cells/well in 2% Matrigel (BD Biosciences) on top of a 100% matrigel layer. Cells were photographed 5 days after seeding. Percentages of invasive cells were defined from more than 400 cell structures. Experiments were performed in duplicate.

### Soft-agar colony assay

To measure anchorage-independent growth, cells were detached with TrypLE-Express and resuspended in growth medium. 6-well plates were prepared with a coating of 0.75% low-melting temperature agarose (50100, Lonza) in complete growth medium and then overlaid with a suspension of cells in 0.45% low-melting agarose (5×10^4^ cells/well). Plates were incubated for 2–3 weeks at 37°C in a humid CO_2_ incubator and colonies were counted under the microscope. Experiments were performed in triplicate.

### Reporter assay

HMEC cells were transfected with 1.2 μg of SNAIL, 0.3 and 0.5 μg of firefly and renilla luciferase reporter constructs using GeneJuice as a transfection reagent (Merck Millipore). 48 h post-transfection, cells were lysed and luciferase activity was measured using the Dual Luciferase Reporter Assay System (Promega). To assess *CDH1* promoter activity, we used luciferase reporter vectors containing either a wild-type *CDH1* promoter fragment (−178, +92 bp) (*CDH1* wt), or the same fragment with mutated E-boxes (*CDH1* mut) [19]. Experiments were performed twice.

## Supporting Information

Figure S1 **Determination by RT-qPCR of** ***SNAI1, SNAI2*** **and** ***SNAI3*** **transcript levels in human mammary cancer cell lines.** Levels expressed relatively to housekeeping gene transcripts were normalized with respect to HMEC-hTERT cells.
(PDF)

Figure S2 **Inefficiency of SNAIL3 in triggering EMT does not rely on protein instability or aberrant subcellular localization.** HMEC cells were infected with constructs encoding C-terminal tagged SNAIL1 or SNAIL3 proteins. (A) Representative photomicrographs of cells obtained by phase contrast microscopy. Note that only *SNAI1* expressing cells underwent an EMT. (B and C) Analysis of SNAIL proteins (anti-HA antibody) and E-cadherin by immunofluorescence. (D) Analysis of epithelial and mesenchymal markers, and of SNAIL proteins by western-blotting. Please note the higher level of SNAIL3 protein. (E) Comparison of the ability of SNAIL1 and SNAIL3 transcription factors to down-modulate the transcriptional activity of a *CDH1*-reporter construct (*CDH1* wt). A reporter harboring mutations in E-boxes was used as a control (*CDH1* mut). Activities normalized with respect to basal reporter activity are indicated ±SD of triplicates.
(PDF)

Table S1 **Expression of EMT-associated genes in SNAIL-HMEC and SNAIL-MCF10A cells.** Expression profiling of genes regulated during EMT transdifferentiation in HMEC-hTERT and MCF10A cells infected with SNAIL1-, SNAIL2-, or SNAIL3- encoding retroviral vectors. Firstly, transcripts of genes reported as downregulated during EMT [42]. Genes downregulated with a fold change (FC) between 1.5 and 3 are labeled in light green; those downregulated with FC>3 are labeled in dark green. Secondly, transcripts of genes reported as induced during EMT [42]. Genes upregulated with 1.5<FC<3 are labeled in orange; genes upregulated with FC>3 are labeled in red.
(PDF)
