# Supplementary figures and images for: Snail Family Members Unequally Trigger EMT and Thereby Differ in Their Ability to Promote the Neoplastic Transformation of Mammary Epithelial Cells
Source: PLoS One. 2014 Mar 17;9(3):e92254. doi: 10.1371/journal.pone.0092254 (PMC3956896; doi:10.1371/journal.pone.0092254)

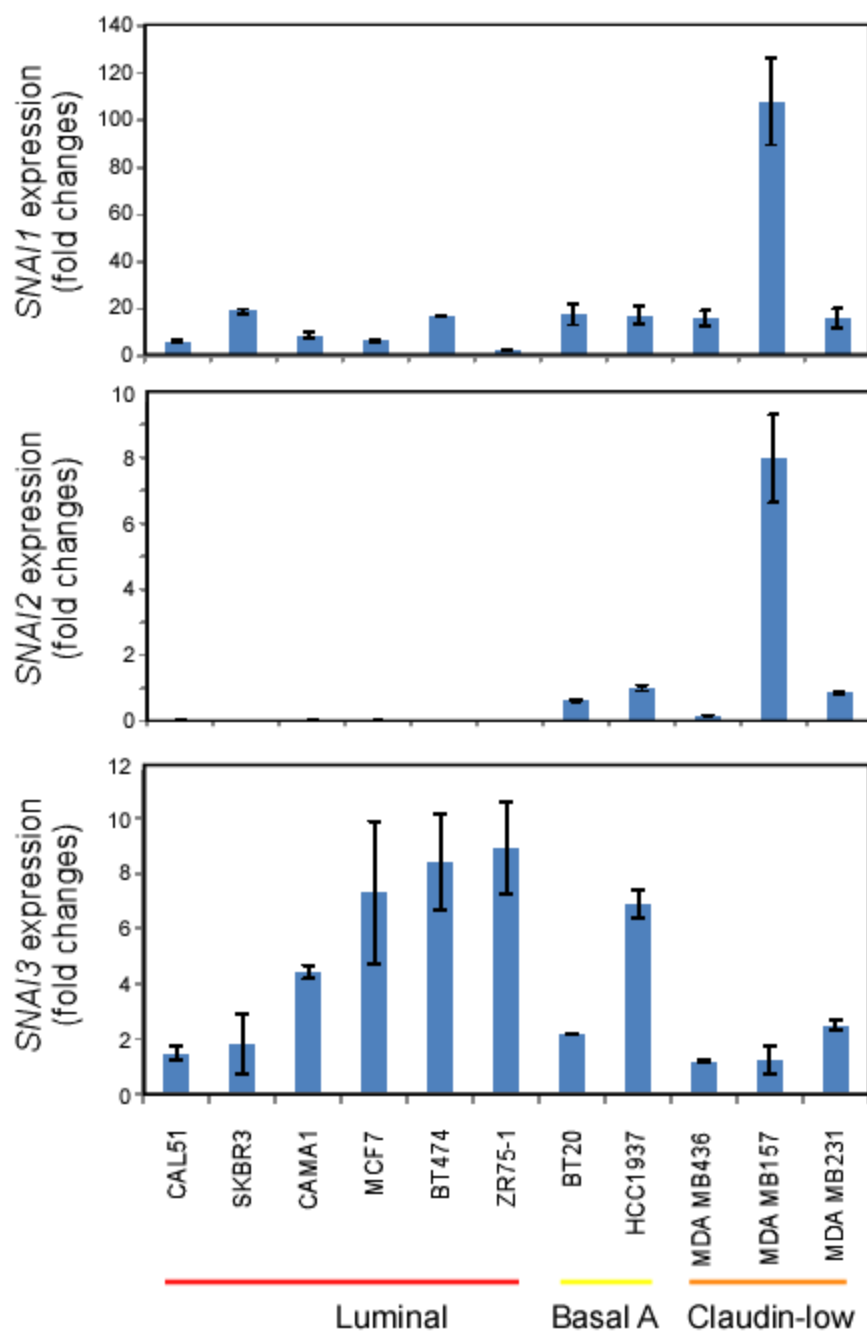

Figure S1

Supplement: Figure S1 — Determination by RT-qPCR of SNAI1, SNAI2 and SNAI3 transcript levels in human mammary cancer cell lines. Levels expressed relatively to housekeeping gene transcripts were normalized with respect to HMEC-hTERT cells. (PDF) [file pone.0092254.s001.pdf]

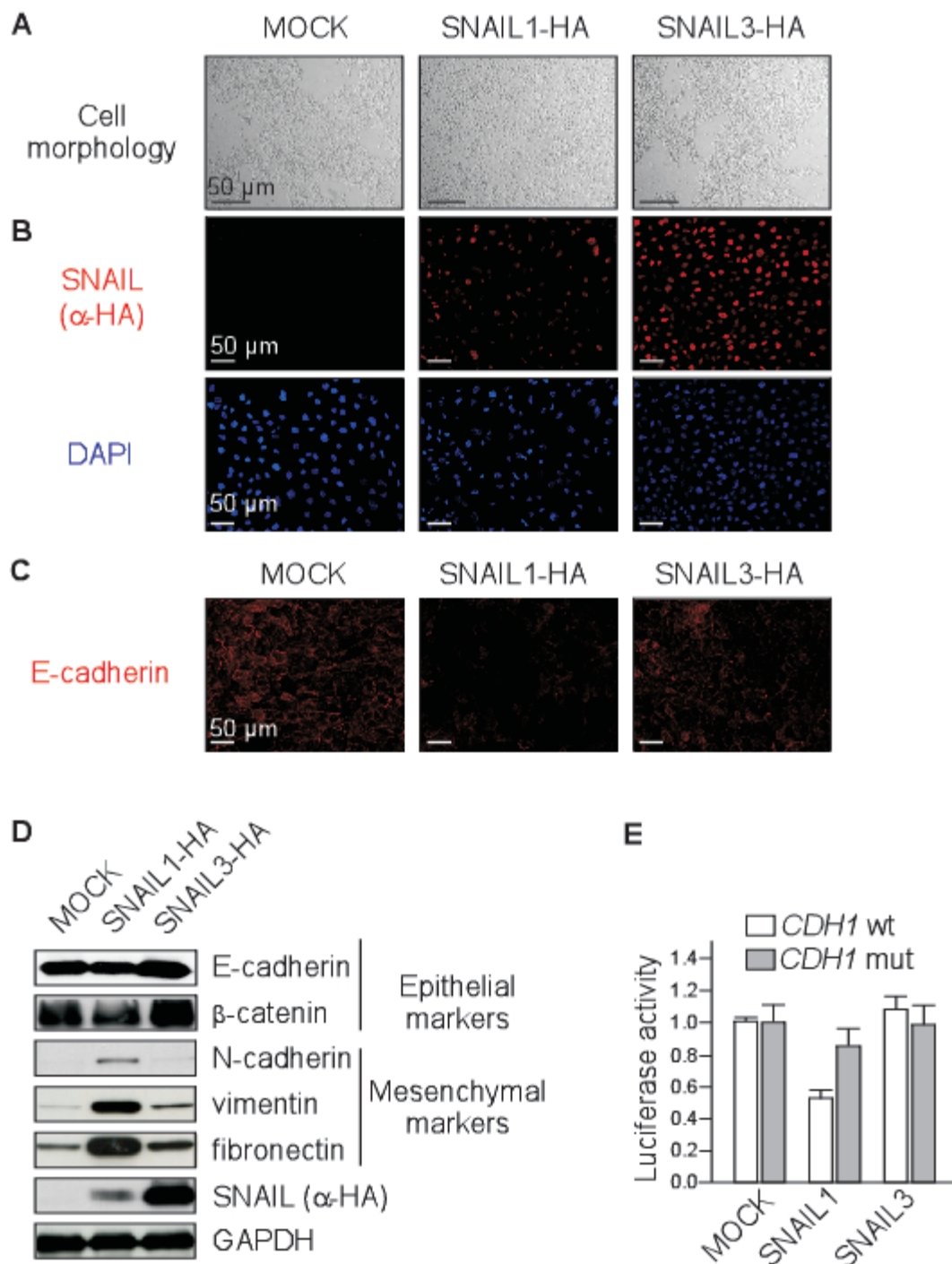

Figure S2

Supplement: Figure S2 — Inefficiency of SNAIL3 in triggering EMT does not rely on protein instability or aberrant subcellular localization. HMEC cells were infected with constructs encoding C-terminal tagged SNAIL1 or SNAIL3 proteins. (A) Representative photomicrographs of cells obtained by phase contrast microscopy. Note that only SNAI1 expressing cells underwent an EMT. (B and C) Analysis of SNAIL proteins (anti-HA antibody) and E-cadherin by immunofluorescence. (D) Analysis of epithelial and mesenchymal markers, and of SNAIL proteins by western-blotting. Please note the higher level of SNAIL3 protein. (E) Comparison of the ability of SNAIL1 and SNAIL3 transcription factors to down-modulate the transcriptional activity of a CDH1-reporter construct (CDH1 wt). A reporter harboring mutations in E-boxes was used as a control (CDH1 mut). Activities normalized with respect to basal reporter activity are indicated ±SD of triplicates. (PDF) [file pone.0092254.s002.pdf]
