# Supplementary material for: Snail Family Members Unequally Trigger EMT and Thereby Differ in Their Ability to Promote the Neoplastic Transformation of Mammary Epithelial Cells
Source: PLoS One. 2014 Mar 17;9(3):e92254. doi: 10.1371/journal.pone.0092254 (PMC3956896; doi:10.1371/journal.pone.0092254)
Supplement: Table S1 — Expression of EMT-associated genes in SNAIL-HMEC and SNAIL-MCF10A cells. Expression profiling of genes regulated during EMT transdifferentiation in HMEC-hTERT and MCF10A cells infected with SNAIL1-, SNAIL2-, or SNAIL3- encoding retroviral vectors. Firstly, transcripts of genes reported as downregulated during EMT [42]. Genes downregulated with a fold change (FC) between 1.5 and 3 are labeled in light green; those downregulated with FC>3 are labeled in dark green. Secondly, transcripts of genes reported as induced during EMT [42]. Genes upregulated with 1.5<FC<3 are labeled in orange; genes upregulated with FC>3 are labeled in red. (PDF) [file pone.0092254.s003.pdf]

Table S1

	HMEC-hTERT SNAIL1		HMEC-hTERT SNAIL2		HMEC-hTERT SNAIL3		MCF10A SNAIL1	MCF10A SNAIL2	MCF10A SNAIL3
Gene	FC	P-value	FC	P-value	FC	P-value	FC	FC	FC
SNX10	-1,61	4,6E-06	-1,03	6,0E-01	-1,01	8,7E-01	-2,10	-1,66	-1,32
TP73L	-2,33	3,0E-03	1,29	1,4E-01	1,32	7,5E-02	-4,20	-2,37	-1,67
KCNK1	-1,55	1,8E-03	-1,12	3,4E-01	-1,09	4,2E-01	-1,44	-1,01	-1,05
BDKRB2	-1,10	3,6E-02	1,00	9,6E-01	-1,04	3,9E-01	-1,17	-1,18	1,03
ANXA8	-1,94	5,1E-04	-1,07	5,9E-01	1,01	9,5E-01	-3,28	-1,66	-1,56
ANXA8L1	-1,70	7,2E-03	-1,18	3,4E-01	1,15	2,7E-01	-2,31	-1,45	-1,37
LOC653562	1,03	2,2E-01	-1,02	5,9E-01	-1,01	7,5E-01	-1,05	-1,10	-1,02
RHBDF2	1,11	1,7E-01	1,18	7,2E-02	1,05	5,2E-01	-1,31	-1,14	-1,07
SLC6A10P	1,32	1,3E-01	1,01	9,7E-01	-1,01	9,7E-01	-1,23	-1,16	-1,06
SLC6A8	1,24	2,7E-01	-1,02	9,4E-01	1,06	8,0E-01	-1,14	-1,03	-1,09
KRT18	-1,27	6,2E-04	-1,08	2,1E-01	1,04	4,4E-01	-1,23	-1,05	-1,11
CDS1	-3,76	9,6E-07	-1,61	1,4E-03	-1,25	3,1E-02	-5,28	-2,37	-1,89
THBD	-1,01	9,8E-01	1,82	1,5E-01	1,44	3,6E-01	-1,49	-1,20	-1,09
NEFM	-1,01	9,0E-01	1,09	2,1E-01	1,12	7,4E-02	1,09	1,15	1,16
RPS6KA1	-1,31	4,2E-03	1,07	3,8E-01	-1,01	8,6E-01	-1,75	-1,38	-1,21
SMPDL3B	-1,62	7,1E-08	-1,54	1,0E-06	-1,17	8,2E-04	-1,66	-1,44	-1,37
ABCA12	-1,40	2,1E-05	-1,34	2,8E-04	-1,15	1,1E-02	-1,26	-1,16	-1,14
RHOD	-1,46	1,1E-04	-1,04	5,7E-01	-1,00	9,9E-01	-1,94	-1,53	-1,38
KRT14	-2,55	5,0E-03	1,01	9,4E-01	1,11	5,6E-01	-1,66	-1,06	1,22
PRKCH	-1,32	2,7E-02	-1,43	2,3E-02	-1,22	1,0E-01	-1,19	-1,29	-1,23
ZBED2	1,07	7,4E-01	1,47	8,5E-02	1,21	3,6E-01	-3,22	-1,72	-1,52
C10ORF10	-1,29	3,8E-01	-1,32	4,3E-01	-1,02	9,5E-01	1,02	-1,01	-1,01
LRRC1	-1,71	8,4E-06	-1,24	1,0E-02	-1,16	3,3E-02	-1,56	-1,34	-1,25
STAP2	-1,51	2,5E-06	-1,15	1,4E-02	-1,09	5,6E-02	-1,17	-1,22	-1,16
JUP	-3,53	6,7E-06	-1,87	1,2E-03	-1,40	1,3E-02	-2,02	-1,57	-1,48
IL4R	-1,70	8,7E-05	1,06	5,0E-01	1,08	3,0E-01	-1,59	-1,38	-1,31
PERP	-2,23	1,2E-03	-1,42	8,0E-02	-1,11	5,0E-01	-2,04	-1,15	-1,30
FGFBP1	-14,84	7,8E-05	-1,41	1,4E-01	-1,07	7,0E-01	-12,23	-2,82	-2,47
MYO1D	-1,25	1,6E-03	1,19	5,7E-03	1,14	2,0E-02	-1,54	-1,28	-1,17
FAT2	-2,59	1,5E-03	-1,19	3,7E-01	-1,05	7,6E-01	-1,86	-1,51	-1,36
WWC1	-2,02	2,6E-06	-1,50	5,0E-04	-1,37	1,1E-03	-2,18	-1,65	-1,29
FZD3	-1,12	9,5E-02	-1,23	2,0E-02	-1,08	2,5E-01	-1,44	-1,36	-1,14
ZNF165	-2,45	2,7E-08	-1,40	1,7E-04	-1,38	9,2E-05	-2,06	-1,87	-1,77
SNCA	-1,31	3,8E-02	1,31	2,4E-02	1,09	4,1E-01	-1,27	-1,53	-1,61
PRSS8	-4,33	3,3E-07	-2,12	6,4E-05	-1,48	1,3E-03	-2,63	-1,63	-1,26
SH2D3A	-1,28	2,0E-02	1,06	5,3E-01	1,10	2,8E-01	-1,53	-1,28	-1,12
GNAL	-1,00	9,7E-01	1,06	7,7E-02	1,02	5,5E-01	1,02	1,05	1,09
BIK	-2,14	1,5E-05	-1,02	8,6E-01	1,05	5,0E-01	-2,85	-1,82	-1,53
CDH3	-3,53	4,8E-06	-1,22	1,1E-01	-1,07	5,0E-01	-3,88	-1,88	-1,42
KIAA0888	1,02	5,3E-01	1,02	6,6E-01	1,08	6,5E-02	-1,01	-1,07	-1,05
KRT5	-3,66	1,6E-03	-1,05	8,2E-01	1,05	8,0E-01	-5,98	-1,80	-1,38
GJB3	-2,09	3,0E-05	1,00	9,9E-01	-1,02	8,1E-01	-2,00	-1,61	-1,39
KIAA0040	-1,69	4,3E-07	-1,17	8,8E-03	-1,10	5,4E-02	-1,20	-1,16	-1,01
CELSR2	-2,40	7,6E-07	-1,73	8,8E-05	-1,34	2,0E-03	-1,68	-1,16	-1,32
F11R	-1,64	1,7E-02	-1,06	7,6E-01	1,01	9,5E-01	-2,19	-1,56	-1,53
NUP62CL	-2,63	2,6E-10	-1,24	1,8E-04	-1,15	1,7E-03	-2,30	-1,44	-1,19
SERPINB1	-4,05	5,4E-07	-3,31	6,5E-06	-1,90	8,0E-05	1,14	-1,01	-1,06
SPINT2	-4,57	5,6E-06	-1,43	2,5E-02	-1,13	3,0E-01	-7,17	-2,69	-1,92
POLR3G	-1,08	4,6E-01	1,03	7,9E-01	1,05	6,7E-01	-1,73	-1,40	-1,32
ELMO3	-1,58	6,5E-03	1,06	6,5E-01	1,03	7,7E-01	-1,45	-1,28	-1,05
IL1RN	-2,07	4,5E-09	-1,89	8,1E-08	-1,39	4,6E-06	1,00	1,01	1,08
TSPAN1	-2,21	2,7E-05	-1,71	1,2E-03	-1,27	2,9E-02	-1,04	1,02	-1,06
IFI30	-1,05	5,0E-01	-1,11	2,6E-01	-1,05	5,6E-01	-1,21	-1,10	-1,05
PLS1	-3,44	2,5E-07	-1,52	9,2E-04	-1,23	1,9E-02	-3,75	-1,75	-1,59
TMPRSS11E	-1,04	2,8E-01	1,03	4,9E-01	-1,02	5,2E-01	1,18	-1,01	-1,01
C1ORF116	-4,86	4,0E-08	-1,72	1,1E-04	-1,34	2,1E-03	-3,61	-2,09	-1,73
ALOX15B	1,50	2,1E-01	1,15	7,5E-01	1,09	8,2E-01	1,29	1,33	1,34
COL17A1	-1,42	1,5E-01	1,25	2,9E-01	1,28	1,9E-01	-3,32	-1,59	-1,39
RTEL1	1,04	2,0E-01	1,08	5,2E-02	-1,05	1,9E-01	-1,05	-1,01	-1,13
TNFRSF6B	1,04	8,0E-01	2,14	1,3E-04	1,77	1,2E-03	-2,13	-1,33	-1,19
LAD1	-2,51	3,3E-05	-1,20	1,5E-01	-1,05	6,2E-01	-5,08	-1,81	-1,39
PTPN3	-1,56	8,2E-09	-1,01	8,0E-01	-1,03	2,2E-01	-1,20	-1,11	-1,01
MST1R	-2,16	3,0E-07	-1,51	1,3E-04	-1,20	8,2E-03	-2,14	-1,50	-1,27
EPHA1	-3,36	2,4E-05	-1,48	2,2E-02	-1,22	1,2E-01	-2,02	-1,47	-1,47
SPINT1	-1,81	2,9E-04	-1,41	1,7E-02	-1,16	1,7E-01	-1,52	-1,32	-1,16
E2F5	-1,83	3,8E-05	-1,53	1,4E-03	-1,28	1,3E-02	-2,22	-1,68	-1,37
KRT6B	-2,99	6,5E-05	1,02	8,5E-01	1,20	1,0E-01	-2,43	-1,76	-1,42
STAC	-1,35	4,6E-03	-1,14	2,0E-01	-1,09	3,0E-01	-1,10	1,15	1,08
ITGB4	-2,04	4,1E-04	-1,06	6,8E-01	1,07	5,5E-01	-1,03	-1,26	-1,19
C6ORF105	-1,09	2,3E-01	1,32	2,4E-03	1,22	1,1E-02	-1,30	-1,09	-1,08
GLS2	-1,28	1,7E-04	-1,12	4,9E-02	-1,09	7,6E-02	-1,23	-1,30	-1,25
ANXA3	-2,51	4,5E-04	-1,02	9,2E-01	-1,06	6,5E-01	-3,68	-1,35	-1,13
DST	-1,33	6,2E-03	1,03	7,2E-01	1,09	2,9E-01	-1,53	-1,38	-1,28
ARHGAP25	-1,07	2,7E-01	1,22	7,0E-03	1,13	5,3E-02	-1,11	-1,05	1,01
DSC2	-2,76	3,9E-02	-1,86	1,8E-01	-1,41	3,4E-01	-2,11	-1,42	-1,38
SLC6A8	1,24	2,7E-01	-1,02	9,4E-01	1,06	8,0E-01	-1,14	-1,03	-1,09
LSR	-1,33	1,0E-06	-1,13	3,2E-03	-1,04	1,7E-01	-1,29	-1,18	-1,19

	HMEC-hTERT SNAIL1		HMEC-hTERT SNAIL2		HMEC-hTERT SNAIL3		MCF10A SNAIL1	MCF10A SNAIL2	MCF10A SNAIL3
**Gene**	**FC**	**P-value**	**FC**	**P-value**	**FC**	**P-value**	**FC**	**FC**	**FC**
CLDN1	-2,99	1,1E-05	-3,80	2,3E-05	-1,95	3,1E-04	-3,33	-2,30	-1,76
CYP4F11	-1,49	3,7E-02	-1,00	1,0E+00	-1,06	7,0E-01	2,31	1,89	1,71
CCND2	-1,10	3,3E-01	1,13	2,6E-01	1,05	6,4E-01	-3,62	-2,11	-1,89
FGFR2	1,02	5,8E-01	1,01	7,0E-01	1,04	2,9E-01	-1,05	-1,04	-1,00
ABLIM1	-2,05	6,8E-06	-1,29	1,4E-02	-1,04	5,9E-01	-1,99	-1,41	-1,35
XDH	-1,55	2,1E-03	1,25	3,6E-02	1,21	4,9E-02	-2,50	-1,61	-1,39
CAMK2B	-1,18	1,0E-02	-1,08	2,3E-01	-1,05	3,9E-01	-1,35	-1,26	-1,31
DSG3	-3,19	1,1E-03	-1,50	1,0E-01	-1,15	4,6E-01	-2,75	-1,80	-1,78
OCLN	-1,03	6,7E-02	-1,06	2,1E-02	-1,03	1,2E-01	1,02	1,01	1,05
KRT17	-1,24	1,4E-01	-1,24	2,1E-01	1,02	9,0E-01	-3,56	-1,77	-1,37
SAA1	-5,58	1,6E-05	-2,56	8,9E-04	-1,27	1,1E-01	-1,98	-1,67	-1,31
PRRG4	-1,58	3,5E-04	-1,36	1,1E-02	-1,17	8,6E-02	-1,29	-1,03	-1,18
ANK3	-1,16	1,6E-01	-1,02	8,6E-01	1,04	6,9E-01	-1,08	-1,18	-1,12
TMPRSS4	-1,50	3,0E-05	-1,47	2,0E-04	-1,38	2,7E-04	1,05	1,04	1,03
CST6	-4,64	1,3E-05	-3,67	1,2E-04	-1,74	3,1E-03	-1,71	-1,44	-1,39
NDRG1	1,72	1,6E-01	1,88	1,5E-01	1,38	4,6E-01	1,48	1,31	1,09
S100A8	-1,35	3,8E-01	-1,32	4,8E-01	1,04	9,1E-01	-1,78	-1,17	-1,06
CORO1A	-2,38	1,9E-04	-1,27	1,2E-01	-1,07	5,8E-01	-1,32	-1,17	-1,08
KLK5	-21,16	1,3E-07	-7,72	2,0E-06	-3,31	5,7E-06	1,00	1,06	1,02
EXPH5	-1,57	5,7E-06	-1,35	5,0E-04	-1,17	1,0E-02	-1,58	-1,61	-1,37
IRX4	-1,08	4,5E-01	-1,22	1,3E-01	-1,11	3,5E-01	1,17	1,18	1,19
IRF6	-3,43	4,1E-05	-1,59	1,5E-02	-1,21	1,6E-01	-2,66	-1,88	-1,64
HOOK1	-3,28	8,1E-08	-1,99	1,3E-05	-1,39	6,3E-04	-2,93	-2,10	-2,00
ARTN	-1,38	3,7E-03	1,05	6,1E-01	1,04	6,2E-01	1,02	-1,05	1,01
FLJ12684	-1,88	2,0E-04	-1,04	7,0E-01	1,01	9,3E-01	-3,06	-1,89	-1,45
SLC2A9	-1,34	2,4E-03	1,15	7,6E-02	1,12	9,9E-02	-1,48	-1,23	-1,16
KLK8	-2,84	1,2E-06	-2,54	1,3E-05	-1,86	5,6E-05	-1,08	1,06	1,01
TMEM40	-1,61	6,0E-04	-1,21	1,0E-01	1,03	7,6E-01	-1,34	-1,24	-1,16
TRIM29	-1,78	1,7E-05	-1,30	8,9E-03	-1,14	7,7E-02	-1,34	-1,35	-1,32
HBEGF	-2,36	5,2E-03	1,45	4,2E-02	1,37	6,2E-02	-1,64	-1,56	-1,46
ALDH1A3	-2,87	5,9E-08	-1,04	5,1E-01	1,01	8,6E-01	-6,76	-3,08	-2,37
MYO5C	-5,93	2,5E-07	-3,81	4,7E-06	-1,82	1,5E-04	-4,34	-2,93	-2,37
CYP27B1	-1,51	1,1E-03	1,50	2,2E-04	1,18	5,2E-02	-4,02	-2,06	-1,68
IL1B	-1,04	9,4E-01	3,35	1,1E-03	2,53	8,4E-03	-3,21	-2,17	-1,50
NMU	-8,36	2,4E-08	-4,20	6,6E-07	-2,34	3,7E-06	-2,63	-1,36	-1,20
KRT16	-1,81	1,2E-01	-1,75	2,1E-01	-1,25	5,0E-01	-1,54	-1,22	-1,16
CDH1	-5,25	2,7E-07	-2,48	2,9E-05	-1,46	2,0E-03	-7,98	-4,19	-3,66
JAG2	-1,97	2,8E-04	-1,27	8,6E-02	-1,01	9,0E-01	-2,08	-1,51	-1,36
VSNL1	-3,29	1,3E-04	-1,46	5,2E-02	-1,36	6,5E-02	-1,64	-1,25	-1,29
RLN2	-1,42	9,4E-08	-1,16	5,5E-04	-1,13	8,6E-04	-1,25	-1,28	-1,08
CTSL2	-3,82	5,5E-06	-2,54	1,6E-04	-1,67	1,6E-03	-1,76	-1,17	-1,09
SYK	-3,08	7,1E-08	-1,54	2,0E-04	-1,18	2,4E-02	-2,10	-1,51	-1,34
SAA1	-5,58	1,6E-05	-2,56	8,9E-04	-1,27	1,1E-01	-1,98	-1,67	-1,31
EPB41L4B	-1,13	1,4E-04	-1,16	1,4E-04	-1,10	1,1E-03	-1,21	-1,12	-1,06
RNF128	-1,06	6,0E-02	1,02	5,2E-01	-1,00	8,9E-01	-1,02	1,13	1,05
ST14	-6,44	1,4E-05	-3,84	2,1E-04	-1,99	1,8E-03	-4,58	-2,90	-2,58
LEPREL1	1,33	7,5E-02	1,38	8,7E-02	1,13	4,8E-01	-1,46	-1,02	-1,05
PI3	-1,89	2,3E-01	-2,29	2,3E-01	1,59	1,7E-01	-1,50	-1,04	1,34
AP1M2	-2,01	7,0E-05	-1,30	3,9E-02	1,05	5,7E-01	-1,36	-1,25	-1,22
CKMT1A	-5,02	4,2E-06	-1,77	2,9E-03	-1,34	3,0E-02	-3,64	-2,01	-1,72
CKMT1B	-3,66	2,8E-06	-1,62	2,7E-03	-1,17	1,2E-01	-3,60	-1,79	-1,73
GRHL2	-2,73	1,3E-07	-1,61	9,8E-05	-1,13	7,5E-02	1,02	1,06	1,00
ARHGAP8	-1,27	7,3E-06	-1,11	8,8E-03	-1,01	6,5E-01	-1,17	-1,07	-1,08
LOC553158	-1,02	7,2E-01	1,00	1,0E+00	1,06	3,3E-01	-1,20	-1,05	1,03
IGFBP2	-1,23	7,7E-01	-1,16	8,6E-01	-1,05	9,5E-01	-1,20	-1,26	-1,27
IL18	-1,07	5,6E-01	-1,08	5,4E-01	1,06	5,8E-01	1,03	1,04	1,06
CA9	1,36	8,4E-01	-1,08	9,7E-01	-1,09	9,7E-01	-1,02	-1,07	-1,02
S100A14	-3,37	1,8E-05	-1,38	3,8E-02	-1,02	8,8E-01	-3,08	-1,58	-1,48
CA2	-2,65	2,1E-02	-1,75	1,5E-01	-1,40	2,7E-01	-2,94	-1,95	-1,69
KRT15	-12,33	4,7E-07	-3,60	2,7E-05	-1,56	2,8E-03	-2,08	-1,58	-1,68
TMEM30B	-3,49	2,1E-07	-1,52	8,2E-04	-1,12	1,4E-01	-1,34	-1,23	-1,09
S100A7	-1,78	3,4E-01	-1,62	4,8E-01	-1,24	6,9E-01	-1,06	1,03	1,07
KLK7	-16,38	8,0E-09	-20,34	4,6E-08	-4,11	1,5E-07	1,14	1,13	1,15
LGALS7	-1,22	3,6E-01	-1,23	4,2E-01	-1,16	5,0E-01	-1,00	1,03	1,01
FST	-11,90	2,6E-04	-1,83	5,1E-02	-1,24	3,1E-01	-1,23	-1,29	-1,11
CXADR	-5,15	5,1E-07	-3,62	8,4E-06	-1,93	1,2E-04	-3,68	-2,77	-3,41
SLPI	-2,36	9,4E-04	-2,33	3,4E-03	-1,16	3,5E-01	-1,43	-1,11	1,06
RBM35A	-2,16	2,1E-07	-1,24	5,8E-03	-1,05	3,3E-01	-1,25	-1,23	-1,23
RAB25	-9,87	2,6E-07	-3,92	9,5E-06	-1,84	2,6E-04	-3,07	-2,37	-2,01
UCHL1	1,12	2,1E-01	1,03	7,7E-01	1,08	4,2E-01	-1,27	-1,15	-1,15
KLK10	-5,73	3,1E-06	-3,89	4,3E-05	-2,27	1,9E-04	-1,03	1,02	1,13
TACSTD1	-11,95	1,7E-07	-4,07	6,8E-06	-1,85	2,1E-04	-13,82	-4,93	-3,90
SERPINB2	-10,65	4,1E-04	-3,52	7,1E-03	-1,81	4,2E-02	-1,93	-1,88	-1,69
SPRR1A	-1,94	7,4E-02	-1,70	1,9E-01	-1,11	7,2E-01	-1,08	-1,12	-1,07
FGFR3	-3,19	2,1E-02	-2,39	8,4E-02	-1,80	1,3E-01	1,34	1,10	-1,02
SPRR1B	-2,25	6,4E-01	-1,69	7,7E-01	-1,42	8,2E-01	-1,19	-1,07	-1,11

	HMEC-hTERT SNAIL1		HMEC-hTERT SNAIL2		HMEC-hTERT SNAIL3		MCF10A SNAIL1	MCF10A SNAIL2	MCF10A SNAIL3
**Gene**	**FC**	**P-value**	**FC**	**P-value**	**FC**	**P-value**	**FC**	**FC**	**FC**
FBLN5	1,15	1,9E-01	1,03	8,4E-01	1,05	6,5E-01	1,95	1,32	1,14
GREM1	4,33	3,2E-01	1,38	9,2E-01	1,03	9,9E-01	-1,11	-1,16	-1,01
COL3A1	-1,06	9,9E-01	-1,08	9,9E-01	-1,08	9,9E-01	1,07	1,05	1,01
COL1A2	1,89	1,4E-01	1,02	9,7E-01	1,01	9,8E-01	-1,05	1,04	1,04
DCN	6,68	1,3E-02	1,59	8,0E-01	1,07	9,7E-01	4,49	2,89	1,90
CDH2	3,34	9,9E-03	1,95	3,1E-01	1,35	6,7E-01	1,16	1,28	1,35
ENPP2	1,27	6,6E-01	-1,07	9,3E-01	-1,08	9,1E-01	1,18	1,13	1,08
POSTN	1,43	2,4E-01	1,14	7,3E-01	1,05	8,9E-01	2,39	1,67	1,20
RGS4	8,39	1,6E-02	1,71	8,2E-01	1,05	9,9E-01	1,01	1,08	1,01
C5ORF13	4,05	9,5E-03	1,57	6,3E-01	1,12	9,1E-01	1,53	1,26	1,41
PRRX1	1,00	1,0E+00	-1,02	9,4E-01	1,02	9,3E-01	1,05	-1,04	1,07
FBN1	2,23	9,3E-05	1,14	5,7E-01	1,12	5,9E-01	1,30	1,12	-1,02
SRGN	1,77	9,1E-05	1,12	4,4E-01	-1,05	7,5E-01	15,85	6,29	2,70
SPOCK1	4,81	3,5E-09	2,41	3,0E-04	1,56	4,2E-02	2,08	1,62	1,50
PRR16	1,67	1,2E-02	1,11	6,8E-01	1,07	7,6E-01	3,10	1,89	1,55
DLC1	1,10	4,4E-02	1,02	7,1E-01	1,06	2,6E-01	1,03	1,03	1,01
BIN1	1,82	1,2E-04	1,11	5,2E-01	1,04	7,8E-01	1,63	1,24	1,20
RGL1	3,24	1,6E-03	1,96	1,6E-01	1,44	4,6E-01	1,84	1,65	1,49
IGFBP4	3,51	9,9E-04	1,68	3,4E-01	1,22	7,2E-01	2,18	1,46	1,33
PVRL3	2,94	6,6E-04	1,68	2,0E-01	1,16	7,2E-01	3,09	1,94	1,63
CDH11	13,50	2,6E-02	4,11	6,0E-01	1,40	9,4E-01	1,13	1,18	-1,00
OLFML3	1,04	3,7E-01	-1,00	9,8E-01	1,01	9,0E-01	1,09	1,01	1,13
MMP2	1,23	1,6E-02	1,17	1,0E-01	1,05	5,9E-01	1,02	-1,11	1,13
MYL9	3,73	3,7E-02	1,25	8,6E-01	1,09	9,5E-01	2,56	1,33	1,07
COL5A2	7,03	5,4E-04	2,36	3,8E-01	1,29	8,3E-01	1,14	1,11	1,11
CTGF	10,19	9,7E-15	4,19	6,3E-09	1,64	4,6E-03	5,01	3,07	2,05
ZEB1	1,26	8,3E-04	1,09	2,3E-01	1,05	4,5E-01	1,22	-1,00	1,10
ROR1	-1,10	4,8E-02	-1,18	1,1E-02	-1,16	1,1E-02	-1,03	1,12	1,05
PTGER2	2,73	2,1E-05	-1,10	7,6E-01	-1,09	7,7E-01	1,19	-1,04	-1,21
CHN1	2,63	1,7E-02	1,32	6,6E-01	1,13	8,4E-01	1,35	1,33	1,28
PMP22	2,79	5,5E-07	1,98	6,1E-04	1,34	9,3E-02	1,22	1,26	1,30
TRAM2	2,61	5,6E-03	1,55	3,5E-01	1,16	7,6E-01	1,72	1,52	1,17
TAGLN	5,47	1,3E-03	1,54	6,8E-01	-1,05	9,7E-01	5,78	3,56	2,17
TNFAIP6	1,49	1,7E-02	1,08	7,0E-01	-1,00	9,8E-01	1,13	-1,09	-1,03
CREB3L1	-1,04	4,7E-01	1,02	7,3E-01	-1,01	8,2E-01	1,12	1,01	1,01
UGDH	1,17	2,1E-02	1,06	4,3E-01	1,01	8,3E-01	1,28	1,13	1,17
HAS2	3,20	1,7E-06	1,69	3,4E-02	1,28	2,9E-01	1,14	1,00	1,06
DNAJB4	3,03	1,1E-02	1,19	8,2E-01	1,05	9,4E-01	1,75	1,37	1,18
CDKN2C	1,13	2,0E-01	1,09	4,5E-01	-1,03	7,7E-01	1,17	1,08	1,00
CCDC92	1,11	3,4E-01	1,01	9,3E-01	1,08	4,9E-01	1,28	1,24	1,35
WNT5A	6,01	8,0E-03	2,61	4,0E-01	1,67	6,9E-01	3,31	2,49	1,75
IGFBP3	1,20	3,3E-01	-2,99	1,5E-02	-1,56	1,1E-01	3,51	2,81	1,83
PPM1D	1,22	8,1E-04	-1,05	4,1E-01	-1,07	2,2E-01	1,27	1,23	1,12
FILIP1L	1,52	3,5E-03	1,12	4,9E-01	1,03	8,7E-01	-1,01	1,01	-1,04
PDGFC	1,87	1,2E-03	1,27	2,9E-01	1,04	8,6E-01	1,45	1,20	1,05
TBX3	1,87	8,1E-06	1,27	6,6E-02	1,07	5,7E-01	1,32	1,14	1,24
XYLT1	1,33	5,2E-07	1,16	1,6E-03	1,02	5,6E-01	-1,04	1,04	-1,02
FAP	2,25	7,0E-04	1,60	9,0E-02	1,31	3,0E-01	1,71	1,60	1,25
DPT	1,09	4,6E-01	1,02	8,7E-01	1,08	5,0E-01	-1,23	-1,19	-1,29
STC1	4,12	9,0E-06	1,95	7,5E-02	1,31	4,9E-01	-1,29	-1,16	1,10
KRT81	1,04	5,3E-01	1,04	6,4E-01	-1,01	8,5E-01	-1,07	-1,04	-1,06
MMP1	1,98	6,6E-05	2,53	5,8E-06	1,92	2,1E-04	-1,00	-1,02	-1,08
HS3ST2	1,00	1,0E+00	1,03	7,5E-01	-1,02	8,5E-01	1,04	1,00	1,05
LMCD1	1,39	1,9E-03	1,04	7,4E-01	1,03	7,7E-01	2,41	1,55	1,16
N-PAC	-1,24	5,0E-03	-1,14	1,0E-01	-1,14	6,3E-02	-1,07	-1,10	1,02
NR2F1	1,25	3,3E-01	-1,36	3,9E-01	-1,26	4,5E-01	4,67	2,59	1,52
SCCPDH	1,01	7,6E-01	-1,11	1,1E-01	-1,09	1,3E-01	1,13	1,05	-1,02
MLPH	3,45	4,4E-05	1,54	2,5E-01	1,10	8,1E-01	1,96	1,79	1,54
LTBP2	1,64	3,8E-05	1,70	7,7E-05	1,43	1,7E-03	1,37	1,23	1,21
TPM1	2,06	6,7E-04	1,14	6,2E-01	-1,02	9,2E-01	2,56	1,50	1,20
DDR2	1,12	3,0E-01	1,05	7,3E-01	1,04	7,7E-01	-1,02	1,02	-1,02
SEMA5A	2,19	8,1E-04	1,21	5,1E-01	1,09	7,4E-01	1,03	-1,03	1,02
TGFB1I1	2,44	1,6E-06	1,84	8,7E-04	1,32	8,0E-02	2,36	1,76	1,46
PCOLCE	1,38	4,8E-02	1,20	3,5E-01	1,13	5,0E-01	1,21	1,18	1,08
STARD13	1,36	1,0E-04	-1,02	7,7E-01	-1,03	6,5E-01	1,45	1,28	1,20
NID1	1,85	5,8E-03	1,08	8,0E-01	1,04	8,8E-01	-1,06	-1,06	-1,06
SYNC1	2,68	4,7E-04	1,45	3,0E-01	1,20	6,1E-01	1,35	1,36	1,28
ENOX1	1,00	1,0E+00	-1,11	2,6E-01	-1,13	1,6E-01	1,21	1,11	1,10
FSTL1	4,10	3,6E-05	2,16	6,1E-02	1,70	1,9E-01	3,12	2,16	1,75
VIM	7,94	1,7E-09	5,75	5,6E-07	2,81	1,1E-03	1,50	1,31	1,36
MME	3,27	2,9E-05	1,21	6,0E-01	1,04	9,2E-01	1,37	1,36	1,28
LTBP1	1,12	1,7E-03	1,04	2,8E-01	-1,02	5,5E-01	1,43	1,07	1,07
NRP1	1,85	2,6E-02	1,38	3,6E-01	1,24	5,1E-01	1,72	1,47	1,45
THY1	4,95	5,6E-04	2,26	2,3E-01	1,60	5,1E-01	1,03	-1,06	-1,09
NEBL	1,39	2,1E-02	-1,39	1,4E-01	-1,21	3,0E-01	1,08	1,06	1,02
TNS3	2,08	4,5E-06	1,36	3,8E-02	1,15	2,9E-01	1,17	1,19	1,25
ECM1	1,07	5,6E-01	-1,02	9,1E-01	-1,06	6,6E-01	1,22	1,23	1,06
FBLN1	1,08	5,2E-01	-1,33	1,2E-01	-1,19	2,5E-01	1,82	1,51	1,26
TUBA1A	-1,04	7,4E-01	1,04	7,7E-01	1,07	5,1E-01	-1,08	-1,14	1,00
COPZ2	1,54	3,1E-05	1,15	1,4E-01	1,05	5,6E-01	1,27	1,07	1,02
CYBRD1	1,94	4,5E-06	1,46	5,7E-03	1,21	1,1E-01	2,35	1,66	1,61
PPAP2B	3,75	9,3E-04	1,15	8,4E-01	-1,02	9,7E-01	1,87	1,44	1,23
PTX3	2,16	2,5E-03	1,15	6,9E-01	1,03	9,3E-01	1,27	1,19	1,21
FADS2	-1,02	7,3E-01	-1,09	3,2E-01	-1,03	7,0E-01	-1,18	-1,19	-1,06
BGN	7,35	2,9E-01	1,65	9,3E-01	1,05	9,9E-01	1,81	1,29	1,14
TSHZ1	1,16	2,5E-02	1,09	2,6E-01	1,04	5,6E-01	1,31	1,14	1,09
ZBTB38	1,41	8,5E-03	1,22	1,7E-01	1,01	9,3E-01	-1,12	-1,08	-1,41
